# P-1931. Health-related quality of life one year after mild COVID-19 infection: a multicenter prospective cohort study

**DOI:** 10.1093/ofid/ofae631.2091

**Published:** 2025-01-29

**Authors:** Marciane Maria Rover, Fernando Scolari, Geraldine Trott, Mariana Dias Silva, Aline Paula Miozzo, Denise de Souza, Rosa da Rosa Minho Santos, Emelyn de Souza Roldao, Gabriela Soares Rech, Raine Fogliati De Carli Schardosim, Duane Mocellin, Jennifer Menna Barreto Souza, Gabrielle Nunes da Silva, Carolina Rothmann Itaqui, Caroline Cabral Robinson, Milena S Marcolino, Bruna Brandão Barreto, Paulo Roberto Schvartzman, Ana Carolina Peçanha Antonio, Maicon Falavigna, Carisi Anne Polanczyk, Andreia Biolo, Regis Goulart Rosa

**Affiliations:** Hospital Moinhos de Vento, Porto Alegre, Rio Grande do Sul, Brazil; Hospital Moinhos de Vento, Porto Alegre, Rio Grande do Sul, Brazil; Hospital Moinhos de Vento, Porto Alegre, Rio Grande do Sul, Brazil; Hospital Moinhos de Vento, Porto Alegre, Rio Grande do Sul, Brazil; Hospital Moinhos de Vento, Porto Alegre, Rio Grande do Sul, Brazil; Hospital Moinhos de Vento, Porto Alegre, Rio Grande do Sul, Brazil; Hospital Moinhos de Vento, Porto Alegre, Rio Grande do Sul, Brazil; Hospital Moinhos de Vento, Porto Alegre, Rio Grande do Sul, Brazil; Hospital Moinhos de Vento, Porto Alegre, Rio Grande do Sul, Brazil; Hospital Moinhos de Vento, Porto Alegre, Rio Grande do Sul, Brazil; Hospital Moinhos de Vento, Porto Alegre, Rio Grande do Sul, Brazil; Hospital Moinhos de Vento, Porto Alegre, Rio Grande do Sul, Brazil; Hospital Moinhos de Vento, Porto Alegre, Rio Grande do Sul, Brazil; Hospital Moinhos de Vento, Porto Alegre, Rio Grande do Sul, Brazil; Hospital Moinhos de Vento, Porto Alegre, Rio Grande do Sul, Brazil; Medical School, Universidade Federal de Minas Gerais, Belo Horizonte, Minas Gerais, Brazil; Bahia Medical School, Universidade Federal da Bahia, Salvador, Bahia, Brazil; Hospital Moinhos de Vento, Porto Alegre, Rio Grande do Sul, Brazil; Hospital de Clínicas de Porto Alegre, Porto Alegre, Rio Grande do Sul, Brazil; Hospital Moinhos de Vento, Porto Alegre, Rio Grande do Sul, Brazil; Hospital Moinhos de Vento, Porto Alegre, Rio Grande do Sul, Brazil; Universidade Federal do Rio Grande do Sul (UFRGS), Porto Alegre, Rio Grande do Sul, Brazil; Hospital Moinhos de Vento, Porto Alegre, Rio Grande do Sul, Brazil

## Abstract

**Background:**

The long-term effects of mild COVID-19 infection on Health-related Quality of Life (HRQoL) are not yet well understood. The study objective was to assess HRQoL and factors associated with its worsening 12 months after mild SARS-CoV-2 infection.

**Figure:**
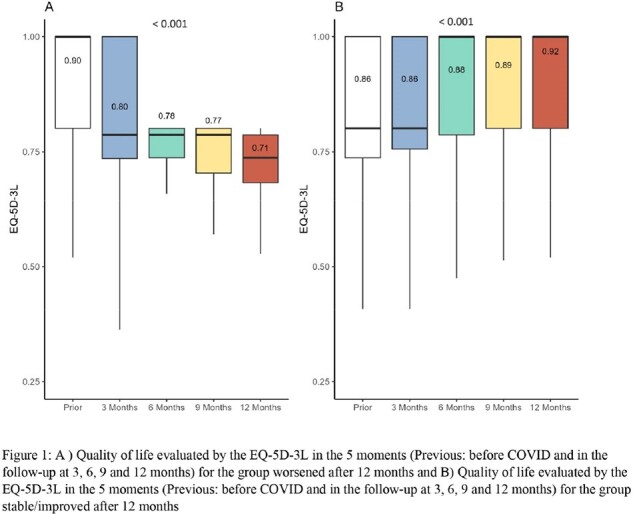

**Methods:**

Multicenter prospective cohort study in Brazil between January 2022 and December 2023. Pasrticipants were ≥ 18 years outpatients for symptomatic SARS-CoV-2 infection confirmed by either RT-PCR or antigen test were included. Telephone interviews conducted at 1, 3, 6, 9 and 12 months after COVID-19 diagnosis. The primary outcome was the HRQoL utility score assessed using the EuroQol-5D-3L (EQ-5D-3L) at 12 months. The patients were categorized into two groups: worsened the EQ-5D-3L and stable/improved in 12 months. Factors associated with worsening or stable/improvement 12 months after COVID-19 were analyzed using Generalized Estimating Equations.

**Figure:**
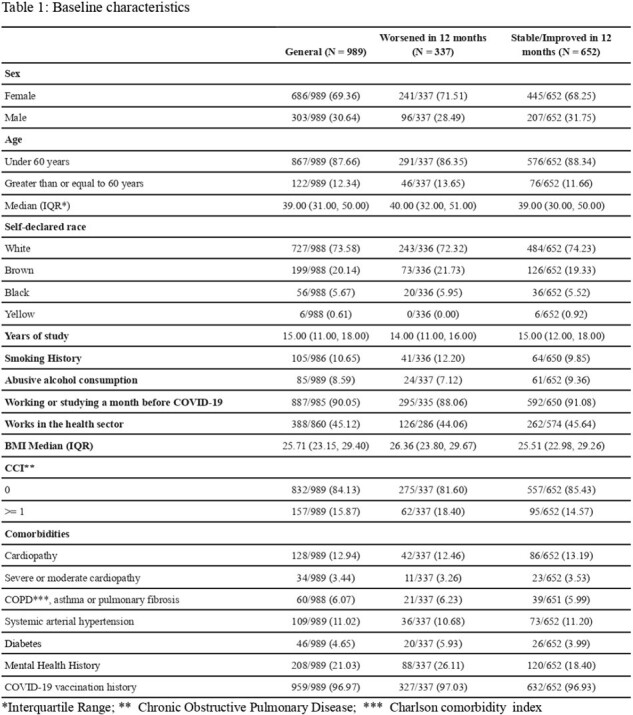

**Results:**

A total of 1174 participants were screened; of these, 1081 were enrolled, and 989 were included in the analysis, with 337 (34%) in the worsened group and 652 (66%) in the stable/imporved group. The median age was 39 (interquartile range, 31-50) years, 69% were women, 45% were health workers, and 84% had no comorbidities (Table 1). In the worsened group, a decline in HRQoL (as measured by EQ-5D-3L with a mean score=0.9) was evident as early as 3 months post- infection (mean score=0.8), being pronounced at 12 months (mean score=0.71, Figure 1). In the stable/improved group, the EQ-5D-3L in 3 months was the same as the previous quality of life (mean score=0.86 in both), with a increase in 12 months (mean score= 0.9, Figure 1). Male sex [0.03 (0.02;0.04)], age 60 and older [-0.03 (-0.04; -0.01)], obesity [-0.04 (-0.05; - 0.03)], Charlson comorbidity Index (CCI) ≥ 1 [-0.05 (-0.06; -0.03)], and history of mental health disease [-0.07 (-0.08;-0.06)] were associated with worsened quality of life at 12 months.

**Conclusion:**

This study shows mild covid-19´s lasting impact on long-term quality of life. The worsened group declined consistently, while the stable/improved group remained stable or improved. Older age, female sex, obesity, and comorbidities or mental health disease were linked to worsened outcomes

**Disclosures:**

Marciane Maria Rover, MD, Hospital Moinhos de Vento: work at Hospital Moinhos de Vento, which received a research grant from the Brazilian Ministry of Health for the conduction of this study. Fernando Scolari, PhD, Hospital Moinhos de Vento: work at Hospital Moinhos de Vento, which received a research grant from the Brazilian Ministry of Health for the conduction of this study. Geraldine Trott, PhD, Hospital Moinhos de Vento: work at Hospital Moinhos de Vento, which received a research grant from the Brazilian Ministry of Health for the conduction of this study. Mariana Motta Dias Silva, MSc, Hospital Moinhos de Vento: work at Hospital Moinhos de Vento, which received a research grant from the Brazilian Ministry of Health for the conduction of this study. Aline Paula Miozzo, PhD, Hospital Moinhos de Vento: work at Hospital Moinhos de Vento, which received a research grant from the Brazilian Ministry of Health for the conduction of this study. Denise de Souza, MSc, Hospital Moinhos de Vento: work at Hospital Moinhos de Vento, which received a research grant from the Brazilian Ministry of Health for the conduction of this study. Rosa da Rosa Minho Santos, n/a, Hospital Moinhos de Vento: work at Hospital Moinhos de Vento, which received a research grant from the Brazilian Ministry of Health for the conduction of this study. Emelyn de Souza Roldao, n/a, Hospital Moinhos de Vento: work at Hospital Moinhos de Vento, which received a research grant from the Brazilian Ministry of Health for the conduction of this study. Gabriela Soares Rech, MSc, Hospital Moinhos de Vento: work at Hospital Moinhos de Vento, which received a research grant from the Brazilian Ministry of Health for the conduction of this study. Raine Fogliati De Carli Schardosim, PhD, Hospital Moinhos de Vento: work at Hospital Moinhos de Vento, which received a research grant from the Brazilian Ministry of Health for the conduction of this study. Duane Mocellin, MSc, Hospital Moinhos de Vento: work at Hospital Moinhos de Vento, which received a research grant from the Brazilian Ministry of Health for the conduction of this study. Jennifer Menna Barreto Souza, n/a, Hospital Moinhos de Vento: work at Hospital Moinhos de Vento, which received a research grant from the Brazilian Ministry of Health for the conduction of this study. Gabrielle Nunes da Silva, PhD, Hospital Moinhos de Vento: work at Hospital Moinhos de Vento, which received a research grant from the Brazilian Ministry of Health for the conduction of this study. Carolina Rothmann Itaqui, n/a, Hospital Moinhos de Vento: work at Hospital Moinhos de Vento, which received a research grant from the Brazilian Ministry of Health for the conduction of this study. Caroline Cabral Robinson, PhD, Hospital Moinhos de Vento: work at Hospital Moinhos de Vento, which received a research grant from the Brazilian Ministry of Health for the conduction of this study. Carisi Anne Polanczyk, PhD, Hospital Moinhos de Vento: work at Hospital Moinhos de Vento, which received a research grant from the Brazilian Ministry of Health for the conduction of this study. Regis Goulart Rosa, MD, PhD, Hospital Moinhos de Vento: work at Hospital Moinhos de Vento, which received a research grant from the Brazilian Ministry of Health for the conduction of this study.

